# Changing medical students’ attitudes to and knowledge of deafness: a mixed methods study

**DOI:** 10.1186/s12909-019-1666-z

**Published:** 2019-06-24

**Authors:** Michelle Gilmore, Anna Sturgeon, Clare Thomson, David Bell, Sophie Ryan, James Bailey, Kieran McGlade, Jayne V. Woodside

**Affiliations:** 1 School of Medicine, Dentistry and Biomedical Sciences, Queen’s University Belfast, Belfast, UK; 2 Southern Health and Social Care Trust, Northern Ireland, UK; 3 Interpreting Works, Belfast, UK; 4 Centre for Public Health, Institute of Clinical Science A, Grosvenor Road, Belfast, BT12 6BJ UK

**Keywords:** Deaf awareness, Deafness, Attitudes, Knowledge, Medical students

## Abstract

**Background and aim:**

Communication with healthcare professionals is challenging for those with hearing loss. This study aimed to determine the impact dedicated deaf awareness training could have on medical student’s attitudes to and knowledge of deafness, and to explore ways of incorporating deaf awareness training into the core undergraduate medical curriculum.

**Methods:**

A validated questionnaire was used to measure attitudes to and knowledge of deafness in those taking an optional deaf awareness and basic sign language module for second year medical students compared to students who took another module. Previous students on this module were also contacted and asked to complete the same questionnaire. Focus groups with these students explored ways to incorporate deaf awareness training into the core undergraduate medical curriculum.

**Results:**

After completing the module, students had a more positive attitude to deaf individuals (*p* < 0.001), and higher knowledge scores (*p* = 0.027) in comparison to the control group. Examination of data revealed a significant negative association between years since undertaking the module and attitudes score (*r* = − 0.29, *p* = 0.04, *n* = 51), with no significant association for knowledge score (*r* = 0.22, *p* = 0.11, *n* = 52). Focus groups suggested integrating deaf awareness training into existing undergraduate communication skills teaching, with the inclusion of deaf tutors.

**Conclusions:**

This study indicates that incorporating a specialist module on deafness can improve attitudes to and knowledge of deafness. Importantly, this effect decreases over time, demonstrating the need for refresher training amongst junior doctors.

## Background

One in six people in the UK are affected by hearing loss. This is estimated to rise to 1 in 5 people affected by 2035, equating to 15.6 million people [1]. Deaf and hard of hearing people frequently encounter difficulties communicating effectively in healthcare settings. Difficulties in accessing healthcare and negative impacts on quality of life are likely to be common for all forms and severities of hearing loss, from those who are hard of hearing but do not need or use a hearing aid, through to those who are profoundly Deaf, are part of the Deaf community and use British Sign Language (BSL). Deaf individuals in the UK receive a substandard degree of care, with the poorer health of deaf British Sign Language users having been attributed to problems in communication and in accessing healthcare [2]: 35% of deaf patients have reported experiencing communication difficulties [3], and 28% haven’t understood their diagnosis after visiting their GP [4].

Doctors also report discomfort in communicating with deaf patients in comparison to their patients in general, particularly in understanding and maintaining conversation; the deaf patients often became frustrated [5], and these encounters then negatively impact on the patient’s trust in their doctor [6]. Doctors are often unaware of the communication needs of these patients, thereby creating barriers and causing feelings of isolation and exclusion.

It is therefore important to provide deaf awareness training for medical students, as they are likely to encounter deaf or hard of hearing patients on a daily basis. However, it is also important that this training is well designed and has an impact on students’ knowledge and attitudes. As part of their training, medical students should be aware of their responsibilities to provide healthcare to all [7]. Training medical professionals to understand hearing loss issues could therefore contribute to broader equality in healthcare. Within the UK and Ireland, two thirds of universities reported that they offered training in deaf awareness and understanding hearing loss, but this was highly variable in terms of the format, the number of students offered it, and the depth of training offered [8].

Since 2002 there has been an optional Student Selected Component (SSC) entitled ‘Sign Language and Communication Tactics’ for second year medical students at Queen’s University Belfast to encourage awareness of the needs of deaf and hard of hearing people, their language and communication. The course was designed in conjunction with Royal National Institute for the Deaf (now Action on Hearing Loss) and QUB medical educators. It currently consists of 17 three-hour British Sign Language (BSL) classes (some of which include role plays, or an invited speaker to talk about healthcare issues for deaf people), 2 deaf awareness sessions and 1 session to develop an online BSL Medical Dictionary which is made available to all medical students. The module runs over a 12-week semester, and students can complete the Signature ‘Introduction to BSL Healthcare Level 101’ examination at the end of this semester. The module won a Signature “Organisational Achievement” award in 2010 [9].

This study aimed to evaluate (i) the impact of specific training on attitudes to and knowledge of deafness, utilising the experience of the Sign Language and Communication Tactics SSC, and (ii) to explore whether any changes in attitudes and knowledge in those who have completed the module persists in the long-term. A third aim (iii) was to explore, using qualitative methods, new ways of further incorporating deaf awareness training into the undergraduate medical curriculum.

## Methods

### *Objective 1:* assess the attitudes of medical students to and knowledge of deaf people before and after completing a ‘Sign Language and Communication Tactics’ SSC

**Fig. 1:**
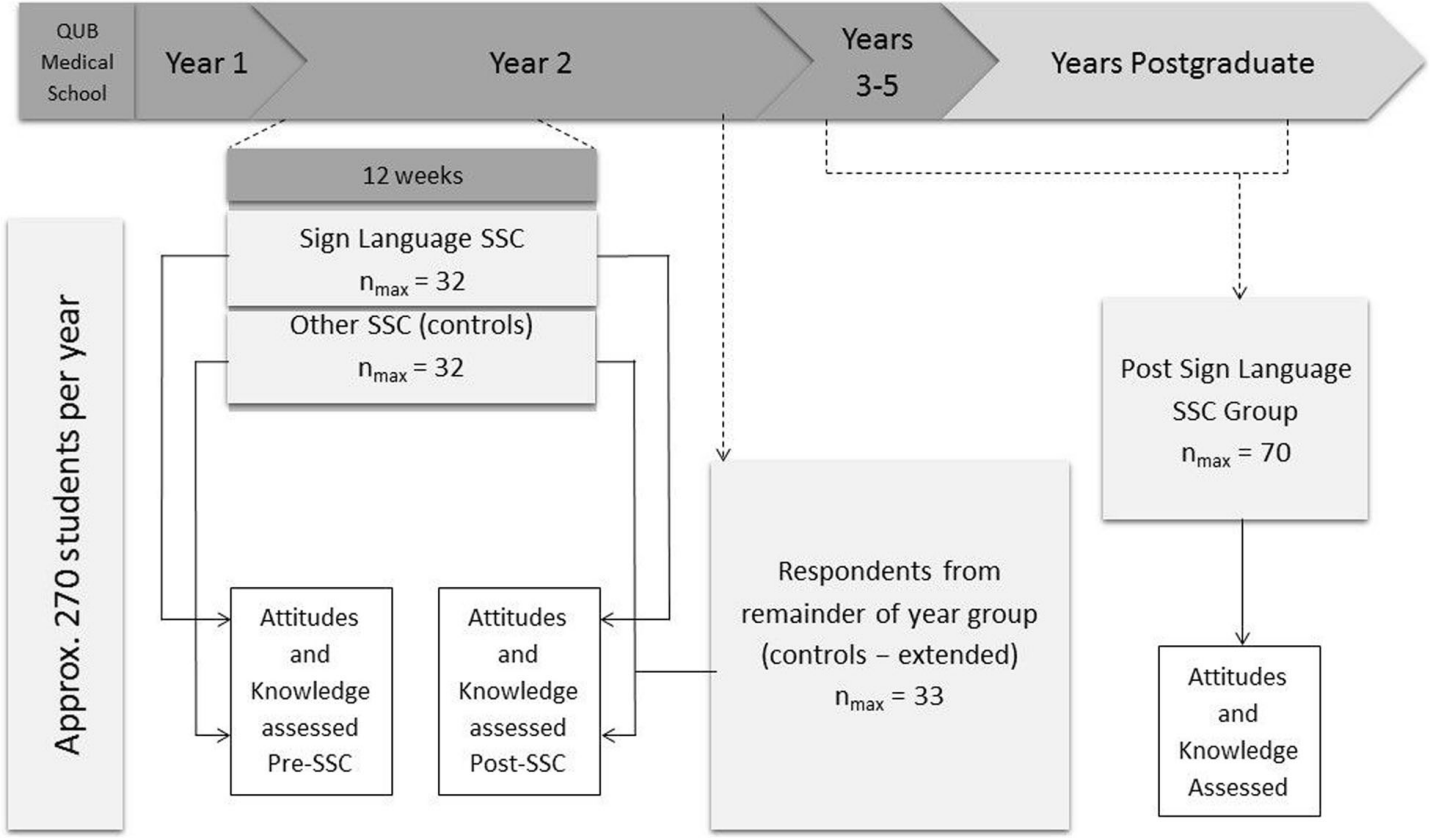
Summary of Study Design

For objective 1, 64 medical students were invited to participate; 32 were in their second year of medical school enrolled in the ‘Sign Language and Communication Tactics’ SSC, while the other 32 were also second year medical students, but who were enrolled in an alternative SSC module, to act as controls. These control SSCs had no overlapping content. The control module was chosen by selecting an SSC of similar size, and the control SSC co-ordinator agreed to their students participating in the study. The remainder of medical students in second year were emailed at the end of the semester to increase the control group numbers available for statistical analysis. Individuals who had already completed the study were removed from this email list to avoid duplicate responses. The study design is summarised in Fig. 1.

The study was explained to all participants, and informed consent obtained. Participants were asked to complete a questionnaire consisting of demographic questions (age, current year of training, participation in SSC, BSL qualifications, graduate or non-graduate entry). Attitudes to and knowledge of deafness was assessed both before and after completing the SSCs. Attitudes to deafness were assessed using the “Attitudes to Deafness Scale” designed by Cooper et al. [10]. This is a 22-item instrument which measures various domains of attitudes towards the deaf, including ability, cultural, equality and linguistic areas [10]. The authors of this scale were contacted regarding permission to use the scale and for advice on scoring the scale. Following discussion with a statistician, the Likert scales were marked 0–5, with the most positive agreement on the scale scoring 5. Knowledge of deafness was measured using a questionnaire which covered a range of questions, including prevalence, aetiology and measurement of deafness [11].

### *Objective 2:* assess the attitudes to and knowledge of deaf people amongst the previous cohorts of medical students and qualified doctors who have previously completed the sign language and communication tactics SSC

Cohorts of students who had previously completed the SSC and who were still enrolled as current medical students were contacted by email and requested to take part in the study. If they were willing they were asked to complete a similar questionnaire, including the attitudes to deafness and knowledge of deafness scales. Previous medical students who had completed the SSC but who had graduated were also contacted. For these participants, approval was sought from the Northern Ireland Medical and Dental Training Agency through Governance procedures following advice from the School of Medicine, Dentistry and Biomedical Sciences Ethics committee. Contact details for doctors who had graduated were accessed through contact details provided by Queen’s University Belfast. Questionnaires were all produced via Survey Monkey and a link emailed to each participant.

### *Objective 3:* explore new ways of incorporating deaf awareness training for all medical students into the core undergraduate medical curriculum

The aim within the University is to develop a programme of deaf awareness training which will be accessible to all medical students (approximately 270 students per year), rather than just the 32 students who currently can take the SSC per year. This may include the development of further online resources from an already existing website which supports the second year SSC. This website includes basic deaf awareness resources and a Medical Sign Language Dictionary. Medical students enrolled in the 2nd year SSC (Sign Language and Communication Tactics) and a smaller 3rd year SSC (Healthcare Issues for the Deaf and Hard of Hearing) were invited to attend a focus group at the end of their SSC to explore the students’ thoughts on the existing modules, the impact on their communication skills, and how further training could be developed and incorporated into the curriculum. All invited students were informed that their participation was voluntary and that the sessions would be audio-recorded. The facilitator was MG, who was undertaking a postgraduate qualification in medical education and was not associated with the courses or their assessment. Focus group sessions were conducted in accordance with standardized protocols, consisting of semi-structured open ended questions in order to ensure a consistent approach between groups. Focus groups were transcribed into Word by MG and any identifiable information was removed from the typed transcripts. The original records were destroyed and the transcripts analysed using thematic analysis.

### Statistical analysis

All statistical analysis was conducted using SPSS, version 22. Data were normally distributed and therefore parametric statistics were used. Assessment of continuous variables between groups was conducted using independent samples t-tests. Association between continuous variables was conducted using Pearson correlation coefficients. A *p*-value less than 0.05 was considered to be statistically significant.

## Results

A total of 29 students undertaking the Sign Language SSC, and 30 students undertaking the control SSC agreed to take part in the study, and the majority these completed assessments both before and after the SSC (*n* = 29 and *n* = 24 respectively), while a further 33 s year students also took part in response to an invitation to participate at the end of semester to enhance the control group. In response to an email request to take part, 70 medical students or doctors who had previously undertaken the Sign Language SSC responded.

### Objective 1

The knowledge score at baseline was similar for the Sign Language and control SSC students. (Table 1). There was, however, a significant difference between Sign Language SSC and control SSC following completion of the module. The students who had completed the Sign Language SSC had higher knowledge scores than those doing the control SSC (*p* = 0.03) (Table 1).

**Table 1:** Effect of a Sign Language module on knowledge of and attitudes to deafness in undergraduate medical students taking either a Sign Language or control SSC

	Knowledge		Attitudes	
Sample	Mean (SD) score pre	Mean (SD) score post	Mean (SD) score pre	Mean (SD) score post
Sign Language SSC	3.0 (1.3) *n 29*	3.7 (1.2) *n 29*	64.6 (8.2) *n 28*	71.0 (10.4) *n 29*
Control SSC	3.1 (0.8) *n 30*	3.0 (1.2) *n 24*	58.6 (7.4) *n 30*	57.0 (8.8) *n 24*
	*p*-value = 0.65^1^	*p*-value = 0.03	*p*-value = 0.005	*p*-value< 0.001
Control SSC (extended)		3.3 (1.25) *n 57*		59.8 (10.2) *n 54*
		*p*-value = 0.15		*p*-value< 0.001

^1^All *p*-values are for comparison of Sign Language SSC versus Control SSC or Control SSC (extended), independent samples t-test

In terms of attitudes, there was a significant difference between the Sign Language and control SSC students at baseline, with participants in the Sign Language SSC having more positive attitudes (*p* < 0.01; Table 1). Similarly, there was a difference in the attitudes score between groups post module, with those doing the Sign Language SSC having more positive attitudes (*p* < 0.001). The magnitude of difference in attitude score between the modules had increased over the course of the module, with the mean difference being 6 points pre-module and 14 points post-module.

When the control SSC data was combined with data from the rest of the year (also post SSC) to form a larger control group, then a significant difference in attitude score, though not knowledge (*p* = 0.15), was confirmed with this larger control group (*p* < 0.001; Table 1).

### Objective 2

There was no significant difference in either knowledge or attitude scores in those who had just completed the SL SSC versus those who had previously completed the SSC (*p* = 0.34 and *p* = 0.88 respectively; Table 2). However, there was a significant negative association between years since undertaking the module and attitudes score (*r* = − 0.29, *p* = 0.04, *n* = 51). There was no significant association for knowledge score (*r* = 0.22, *p* = 0.11, *n* = 52). [Table 2].

**Table 2:** Effect of a Sign Language module on knowledge of and attitudes to deafness in undergraduate medical students recently completing the module versus those who had previously completed the module

Sample	Knowledge				Attitudes			
	N	Mean (SD) score immediately post SSC	N	Mean (SD) score in those previously completing the module	N	Mean (SD) score immediately post SSC	N	Mean (SD) score in those previously completing the module
Sign Language SSC	29	3.7 (1.2)	70	3.4 (1.3)	29	71.0 (10.4)	69	70.6 (10.5)
	*p*-value = 0.34				*p*-value = 0.88			

### Objective 3

Transcripts were available from two focus groups, one group from the second year SSC, and one group from third year SSCA number of themes predominated, and these are described by identified theme below, with representative quotes in Table 3.

**Table 3:** Emergent main themes, sub-themes and illustrative quotes (Q)

Theme	Sub-theme	Quote
Misunderstandings of people with hearing loss	Uncertainty	“At the start of the module I wasn’t really all that sure about what would be classified as deaf.” (Q1)
		“My knowledge was basically that deafness was loss of hearing which usually came with old age.” (Q2)
	Miscommunication	“You would probably think they would be an awkward patient because it’s someone that it’s difficult to communicate with” (Q3)
		“If you saw deaf people in public behaving excitedly or being particularly expressive this would be perceived as ‘not normal’.” (Q4)
Predominance of the medical model	Defining disability	“The hearing family see it as like a disease they would like to fix or find a cure for whereas deaf families have their culture and their pride and things like that and it’s not really a disability” (Q5)
	Application of model	“The purpose would be to treat someone’s pathology or problem they have underlying. The medical person themselves sees that as a problem, the deaf person themselves doesn’t see that as a problem” (Q6)
		“… you could offend a deaf person in implying that deafness is an affliction when some people are happy being deaf.” (Q7)
Exclusivity of deaf culture	Experience	“Unless you actually meet a deaf person then you don’t have a full understanding. I had never had any contact with a deaf person until this project. There are many groups of people with whom unless you have a direct relationship with them, you are not going to be able to identify what their issues are.” (Q8)
		“[the deaf tutor] said ‘nobody is going to get more than 3 [grade], because if you’re not deaf, you can’t understand it.’” (Q9)
	Pride	“There seems to be a certain amount of pride that comes with the deaf community than maybe you hear about with lots of other disabilities … a lot of people wouldn’t want to change it [their deafness]” (Q10)
Best teaching practice	Interactive tutorials	“make it interactive. Because I think if you are actually learning some signs and coming away with something you can do practically, that’d be useful.” (Q11) “I thought it [existing material] was very similar to our communication skills videos we would get during our semesters, I thought it was useful in that way, showing good practice and bad practice at the same time so you can contrast the two.” (Q12) “incorporate it into the system and give value to it, for example if you don’t attend this tutorial you won’t get 20%” (Q13)
	Deaf person involvement	“you could … ask him [the deaf tutor] to come in and show the differences [good practice and bad practice communicating with a deaf patient]” (Q14)
	Videos preferred	“It’s really hard to convey a 3D picture in 2D and like show the motion in two separate stages, you kind of need to show the whole sequence. Video is a lot better than the pictures” (Q15)

#### Misunderstandings of people with hearing loss

Students described uncertainty surrounding what deafness meant before the SSC, and a lack of exposure to deaf people outside the SSC (Q1–2). They described the potential for doctors without knowledge of deafness to be uncomfortable communicating with deaf patients (Q3). Students discussed that a lack of deaf awareness training could inadvertently lead to the mannerisms of deaf individuals being considered rude e.g. pointing gestures, stamping foot to gain attention (Q4).

#### Predominance of the medical model

Students felt that the medical model continues to predominate and discussed limitations in this approach. They considered how the medical model may lead to difficulties in hearing families with a deaf child (Q5), and discord between the doctor and patient (Q6). They were knowledgeable about causes of deafness, and described how healthcare professionals might incorrectly assume that all deaf individuals would wish to seek treatment (Q7).

#### Exclusivity of deaf culture

There was a consensus that it was difficult to truly understand Deaf culture unless one is deaf. They described how many individuals are proud to belong to the deaf community and recognized that, without encountering deaf people, it may prove difficult to understand the issues facing them (Q8–10).

#### Best teaching practice

With regards to the development of material for use by the broader undergraduate medical student population, it was advised that this should be interactive and user-friendly (Q11). They reported that material showing both good practice and bad practice was beneficial (Q12). Students felt it was important to increase awareness of material that is already in existence and suggested that this could occur within a tutorial, which would be led by a deaf person with experience of teaching. They also reported that material needed to have a value (i.e. be assessed in some way) for students to engage (Q13). Students suggested that it would be valuable to integrate deaf awareness training with the currently offered communication skills training, as there would be significant overlap, and it would also be useful if a sign language interpreter was involved in these tutorials (Q14). Students did not feel distributing a leaflet or an email about deaf awareness and basic sign language would be beneficial, rather a lecture or tutorial with face-to-face delivery would be preferable (Q15).

## Discussion

This study aimed to explore the effect of deaf awareness and basic sign language training offered to second year medical students as an SSC on knowledge of and attitudes to deafness in the short and longer term and also to explore how best to extend formal deaf awareness training to the full cohort of undergraduate medical students.

It was anticipated that both Sign Language SSC students and control students would score similarly at baseline for knowledge. The finding that knowledge scores increased following completion of the module for only the Sign Language SSC was unexpected, as students are not directly lectured on the information collected within the questionnaire. Possible reasons for this may be that, over the course of the module students either gain this information through interactions with their tutor or by accessing this information themselves outside the module. However, broadening the control group at the end of semester to include the rest of the year group, and including these data in the analysis led to the difference in knowledge between those completing the Sign Language SSC and those not being not significant. Only 33 students responded to the email to participate out of approximately 210 who would have been eligible to participate, therefore the representativeness of this group in terms of their knowledge of and attitudes to deafness is uncertain.

Examination of the data revealed that, at baseline, when comparing Sign Language SSC students and the control SSC students the Sign language group had a more positive attitude to deafness. Students self-select to participate in the SSC and it may have been anticipated that students who ranked the SSC highly in their choices would have had an interest in this area. This is particularly the case, considering it is a contact-heavy module, which can affect module popularity. Equally, the finding that the magnitude of difference between the two groups increased after completion of the module might also be anticipated, as studies have shown that contact with a particular group (e.g. deaf people, religious groups, psychiatrists) appears to improve attitudes toward that particular group [12, 13].

The mean knowledge score of those who had previously completed the module was 3.4, which was not statistically significantly different from those recently completing the module (3.7). Similarly, there was no statistically significant difference in attitudes in those recently completing the module versus those who had completed it in the past. If one assumes that these participants would also have scored a similar baseline this would indicate that some level of knowledge is retained despite a number of years since completion of the module. However, the finding that the attitudes results were significantly negatively associated with years since completion of the module would indicate that the beneficial effects of the module on attitudes to deafness may reduce as time since completing the module extends, and may also suggest a need for updated deaf awareness training. This result is similar to attitudinal results in studies in other settings, and this effect has been referred to as decaying [14], which may be related to changes in empathy [15]. It must also be taken into consideration that the individuals in the previous cohort group who completed the questionnaire are likely to be a more motivated group and one cannot therefore assume that the individuals who failed to respond would have scored similarly.

With regards to creating teaching material to allow involvement of all undergraduates, the focus groups generated a number of key ideas. Potential improvements suggested involved the inclusion of resources available in the public domain. Limitations of the website mentioned included the need for high speed internet and also the lack of specific medical terminology signs within the online medical dictionary generated by the students. In Northern Ireland, colloquialisms exist and Northern Irish signs will differ from the BSL signs used in other resources. This will be challenging to deal with on an ongoing basis. Importantly, communication must be two way and the ability to sign a few words is not in any way a replacement for accessing an interpreter, therefore inclusion of training regarding accessing interpreters will be essential in any educational material. Consideration must be given to the hidden educational agenda, which is a factor in undergraduate education [16]. This refers to the preconceived areas which students have identified as essential in their studying. Students were clear that without an examination or goal of some sort students were unlikely to access information in regards to deaf awareness.

Students suggested incorporating deaf awareness teaching into ongoing communication skills training, potentially with a deaf teacher and sign language interpreter leading a tutorial combined with the already available online material as a multi-modal approach. Deafness could be used as an example where communication can be challenging, therefore such an inclusion would be logical. However consideration must be given to constraints, as there would be resource implications in terms of funding, suitable tutors and provision of interpreter support where required. Key messages and information to be included within a short time period (maximum 2 h) would have to be considered carefully. Deaf awareness training provision is currently variable in medical schools, with little consistency in scope of training, and whether it is optional or compulsory [8] but, given that communication with healthcare professionals is challenging for those who are Deaf or hard of hearing [2–4], and that guidance exists [17], it would appear prudent for medical schools to consider deaf awareness training/communication training provision.

## Conclusion

This study has described the impact that dedicated deaf awareness training can have on the attitudes to and knowledge of deafness amongst medical students. The Sign Language Student Selected Component studied was an effective mechanism for improving attitudes to deaf individuals and improving student’s knowledge, and indicated the value of dedicated deaf awareness training in the undergraduate curriculum, although regular refresher training may be required. Students were positive re: the inclusion of deaf awareness training for the wider student population, although such an extension requires consideration of scope, appropriate development and piloting to test effectiveness.

## Data Availability

The datasets generated and analysed during the current study are available from the corresponding author on reasonable request.

## Abbreviations

BSL: British Sign Language
SSC: Student Selected Component

## References

[1] Action on Hearing Loss. 2016. https://www.actiononhearingloss.org.uk/about-us/our-research-and-evidence/facts-and-figures/ Accessed 24 Sept 2018.

[2] SignHealth. 2013. https://www.signhealth.org.uk/sick-of-it-report-professionals/ Accessed 24 Sept 2018.

[3] Royal National Institute for the Deaf [RNID] (2004). A Simple Cure: A national report into deaf and hard of hearing peoples’ experience of the National Health Service.

[4] Action on Hearing Loss [AOHL] (2015). Hearing Matters: Why urgent action is needed on deafness, tinnitus and hearing loss across the UK.

[5] Andrade PC, De Carvalho Fortes P, De Carvalho Fortes PA (2010). Communication and information barriers to health care assistance for deaf patients. Am Ann Deaf.

[6] Emond A, Ridd M, Sutherland H, Allsop L, Alexander A, Kyle J (2015). Access to primary care affects the health of deaf people. Br J Gen Pract.

[7] Northern Ireland Government (2006). The Disability Discrimination (Northern Ireland) Order.

[8] McGlade K, Saunders E, Thomson C, Woodside JV (2013). Deaf awareness training in medical schools. Med Teach.

[9] McGlade K, Woodside J. Teaching deaf awareness within healthcare disciplines. http://www.med.qub.ac.uk/DeafAwareHealth/index.html Accessed 24 Sept 2018.

[10] Cooper S, Rose J, Mason O (2004). Measuring the attitudes of human service professionals toward deafness. Am Ann Deaf.

[11] Cooper A, Rose J, Mason O (2003). Mental health professionals ‘attitudes towards people who are deaf. J Community Appl Soc Psychol.

[12] Allport GW (1954). The nature of prejudice.

[13] Pettigrew T (1998). Intergroup contact theory. Annu Rev Psychol.

[14] Cook PD, Flay BR, Berkowitz L (1978). The persistence of experimentally induced attitude change. Advances in Experimental Social Psychology.

[15] Hojat M, Mangione S, Nasca TJ, Rattner S, Erdmann JB, Gonnella JS, Magee M (2004). An empirical study of decline in empathy in medical school. Med Educ.

[16] Nunan D, Breen M, Johnson RK (1989). Classroom implementation. The second language curriculum.

[17] Middleton A, Niruban A, Girling G, Myint PK (2010). Communicating in a healthcare setting with people who have hearing loss. BMJ.

